# Announcement: 9th International Conference on Biological Inorganic Chemistry, ICBIC 9

**DOI:** 10.1155/MBD.1998.321

**Published:** 1998


					ICBIC 9
9TH INTERNATIONAL CONFERENCE
ON BIOLOGICAL INORGANIC CHEMISTRY
July 11-16 1999
Minneapolis, Minnesota USA
Contact Person" Judy Sunvold
Professional Education Programs
405 Coffrey Ha11-1420 Eckles Ave., St. Paul, MN 55108-6068, USA
612-6253775-phone; jsunvold @ extension.umn.edu
Conference Web site"
http://ICBIC9.chem.umn.edu/
Organizing Committee
Lawrence Que, Jr.,Chair Marian T. Stankovich
John D. Lipscomb William B. Tolman
Douglas H. Ohlendorf Lawrence P.Wackett
Nancy Hagberg, Executive Secretary
(ICBIC9 @chem.umn.edu)
Those planning to attend the conference are requested to contact Judy Sunvold.
This will ensure receipt of the second circular. This second circular, containing
further details, abstract information, and the final registration will be available in
the fall of 1998
The "Twin Cities" of Minneapolis and St. Paul is an exciting and progressive
community set along the scenic Mississippi River. Endowed with friendly people
and a clean environment, the area draws many visitors during the summer
months. With a 72 precent chance of sunny weather, July isa great time to
experience the area's lakes and parks, along with the summer's boundless
outdoor recreational and cultural activities. The average high temperature of 83 F
and the average low of 64F make for pleasant days and comfortable nights.
321
Visitors will experience a thriving arts community which includes performances
by the internationally acclaimed Gurthie Theather and the Minnesota Orchestra:
highlights for museum goers include the Walker Art Center and Sculpture
Garden, The Minneapolis Institute of Arts, The Weisman Art Museum, The
Science Museum of Minnesota, and the Minnesota History Center, and people of
all ages gravitate to the Minnesota Zoo and the Minnesota Twins and St. Paul
Saints baseball teams. World-class shopping is available in both downtowns, in
many smaller shops throughout the twin Cities, and at the Mall of America-the
largest indoor shopping mall in the U.S.
Topics
Bioenergetics
Nitrogen and Sulfur Metabolism
Oxygen Activation
Photosynthesis
C-H Bond Activation
Biodegradation
Macromolecular Cleavage
Sensing, Signaling and Gene Regulation
Biotechnologic Applications
Spectroscopic Methods
Reactive Intermediates
Plenary Lecturers
Prof. Helmut Beinert, University of Wisconsin, Madison
Prof. Lucia Banci, University of Florence
Prof. Eiichi Kimura, Hiroshima University
Prof. Judith P. Klinman, University of California, Berkeley
Prof. Edward I. Solomon, Stanford University
Prof. Karl Wieghardt, Max-Planck-lnstitut, Melheim
322

				

